# Magnetotactic Bacteria as Potential Sources of Bioproducts

**DOI:** 10.3390/md13010389

**Published:** 2015-01-16

**Authors:** Ana Carolina V. Araujo, Fernanda Abreu, Karen Tavares Silva, Dennis A. Bazylinski, Ulysses Lins

**Affiliations:** 1 Instituto de Microbiologia Paulo de Góes, Universidade Federal do Rio de Janeiro, Avenida Carlos Chagas Filho, 373, CCS, UFRJ, Rio de Janeiro, RJ 21941-902, Brazil; E-Mails: acvaraujo@gmail.com (A.C.V.A.); fernandaaabreu@micro.ufrj.br (F.A.); tsilvakaren@gmail.com (K.T.S.); 2 Ludwig-Maximilians-Universität München, Department of Biology I, Microbiology 82152, Planegg-Martinsried, Germany; 3 School of Life Sciences, University of Nevada at Las Vegas, Las Vegas, NV 89154-4004, USA; E-Mail: Dennis.Bazylinski@unlv.edu

**Keywords:** biomineralization, bioproducts, genome mining, greigite, magnetite, magnetosomes, magnetotactic bacteria, *Magnetovibrio blakemorei*, nonribosomal peptide synthetase, polyketide synthase

## Abstract

Magnetotactic bacteria (MTB) produce intracellular organelles called magnetosomes which are magnetic nanoparticles composed of magnetite (Fe_3_O_4_) or greigite (Fe_3_S_4_) enveloped by a lipid bilayer. The synthesis of a magnetosome is through a genetically controlled process in which the bacterium has control over the composition, direction of crystal growth, and the size and shape of the mineral crystal. As a result of this control, magnetosomes have narrow and uniform size ranges, relatively specific magnetic and crystalline properties, and an enveloping biological membrane. These features are not observed in magnetic particles produced abiotically and thus magnetosomes are of great interest in biotechnology. Most currently described MTB have been isolated from saline or brackish environments and the availability of their genomes has contributed to a better understanding and culturing of these fastidious microorganisms. Moreover, genome sequences have allowed researchers to study genes related to magnetosome production for the synthesis of magnetic particles for use in future commercial and medical applications. Here, we review the current information on the biology of MTB and apply, for the first time, a genome mining strategy on these microorganisms to search for secondary metabolite synthesis genes. More specifically, we discovered that the genome of the cultured MTB *Magnetovibrio blakemorei*, among other MTB, contains several metabolic pathways for the synthesis of secondary metabolites and other compounds, thereby raising the possibility of the co-production of new bioactive molecules along with magnetosomes by this species.

## 1. Introduction

Magnetotactic bacteria (MTB) are a morphologically, phylogenetically, and metabolically diverse group of prokaryotes that share the ability to biomineralize intracellular magnetic nanocrystals surrounded by a lipid bilayer (biological membrane) [1]. These structures, referred to as magnetosomes, are usually organized in one or more chains within the cell, function as a miniature biological compass needle thereby causing cells of MTB to orient and subsequently swim along magnetic field lines [1]. Magnetosome crystals are either composed of the iron oxide, magnetite (Fe_3_O_4_), or the iron sulfide, greigite (Fe_3_S_4_), depending on the species. Usually one species of MTB synthesizes magnetosome crystals of one specific composition, magnetite or greigite, although several species synthesize both minerals [2,3]. MTB mainly occur in aquatic ecosystems ranging from freshwater to hypersaline environments. Most MTB are microaerophilic with regard to oxygen, although there are some obligately anaerobic strains usually found at or below the oxic-anoxic interface of chemically stratified water columns or sediments [1]. While magnetite-producing MTB are generally found at the oxic-anoxic interface, greigite synthesizers inhabit more anoxic zones where sulfide is present [4].

All MTB described so far phylogenetically belong to the domain *Bacteria*. Most species belong to several classes in the *Proteobacteria* phylum including the *Alpha-*, *Delta-* and *Gammaproteobacteria* [5,6,7]. A number of uncultured MTB belonging to the *Nitrospirae* phylum and the *Planctomycetes-Verrucomicrobia-Chlamydiae* (PVC) superphylum have also been described [8,9,10]. Cell morphologies of MTB include cocci, rods, spirilla, vibrios, barbell-shaped and multicellular forms. Despite their phylogenetic and morphological diversity, relatively few MTB are currently maintained in axenic cultures.

Recognized cultured spirillar MTB belonging to the genus *Magnetospirillum* in the *Alphaproteobacteria* were all isolated from freshwater habitats: the most studied species of this genus include *Ms. gryphiswaldense* [11], *Ms. magneticum* [12], *Ms. magnetotacticum* [13]. Isolated strains from marine or brackish environments include: the coccoid strains MO-1 [14], *Magnetococcus marinus* strain MC-1 [15] and *Magnetofaba australis* (IT-1) [16]; the vibrioid strain *Magnetovibrio blakemorei* strain MV-1 [17] and the spirilla *Magnetospira thiophila* (MMS-1) [18] and *Magnetospira* sp. strain QH-2 [19]; all of which belong to the *Alphaproteobacteria*. Deltaproteobacterial MTB found in marine habitats include the multicellular forms: *Candidatus* Magnetoglobus multicellularis, from a hypersaline lagoon connected to the sea [20]; *Candidatus* Magnetananas tsingtaoensis from an intertidal zone [21]; and *Candidatus* Magnetomorum litorale from the North Sea [22]. Deltaproteobacterial MTB also include the freshwater *Desulfovibrio magneticus* strain RS-1 [23] and a species from a brackish environment *Candidatus* Desulfamplus magnetomortis strain BW-1 [24]. There are two species of cultivated MTB belonging to the *Gammaproteobacteria*, strains BW-2 and SS-5, isolated from a brackish and a hypersaline environment, respectively [25].

The composition and morphology of magnetosome crystals is species specific and is strongly correlated with the phylogeny of MTB [26], evidence that there is strong genetic control involved in magnetosome biomineralization. Each magnetosome consists of a magnetic crystal surrounded by a lipid bilayer that originates from the cell (cytoplasmic) membrane, but has a different protein composition [27]. Several proteins, considered unique to MTB, are located in or close to the magnetosome membrane (MM) and appear to control crystal nucleation, growth, and the organization of magnetosomes within the cell [28]. Each species has control over the composition, direction of crystal growth in elongated particles, and the size and shape of its own magnetosome mineral crystals. However, the local environment clearly influences magnetosome synthesis since it has been shown in culture, for example, that different concentrations of oxygen and iron affect magnetosome composition, crystal size, and crystallographic properties [29,30,31].

Biogenically-produced magnetosomes present unique features that are difficult to obtain through the chemical synthesis of abiotically-produced magnetic nanocrystals. These characteristics include: a narrow, single magnetic domain, nanosize range; a strong degree of crystallographic perfection; a permanent magnetization; and the presence of a biocompatible lipid bilayer around each mineral particle [32,33]. All these characteristics have outstanding importance in biotechnological applications of magnetic nanoparticles such as contrast for nuclear magnetic resonance (NMR), in cell separation assays, as drug carriers and in the destruction of tumor cells by hyperthermia [34,35,36,37].

The main shortcoming for the application of magnetosomes is the requirement for large amounts of material through the mass culture of MTB. Although in general MTB are fastidious with regard to growth, the cultivation in large bioreactors has already been established for freshwater strains of *Magnetospirillum* [38] and the marine vibrio *Magnetovibrio blakemorei* [39]. The major goal is to increase magnetosome production while decreasing the cost of the cultivation of MTB. One methodology to avoid this problem is to transfer the genetic capability to biomineralize magnetosomes to a more easily-grown non-magnetotactic bacterium. This has now been established in the photosynthetic alphaproteobacterium *Rhodospirillum rubrum* [40] although large-scale production of magnetosomes by heterologous expression in this organism has not yet been tested. Another strategy to increase the feasibility of the industrial production of magnetosomes is to co-produce compounds of high added-value in the process since the magnetic properties of the nanocrystals facilitate their separation. The availability of a number of genomes from MTB has enabled searches for genes encoding biosynthetic pathways not detected under currently applied culture conditions.

Here, we review recently acquired knowledge regarding MTB, magnetosome biomineralization, and their mass production in bioreactors and technological applications where magnetosomes might be superior compared to currently used materials. We also searched for conserved domains of genes in the genomes of MTB that encode the production of secondary metabolites thereby targeting strains of MTB with the potential to produce high added-value compounds.

## 2. Biology of MTB and Their Magnetosomes

### 2.1. Ecology and Physiology of MTB

**Figure 1:**
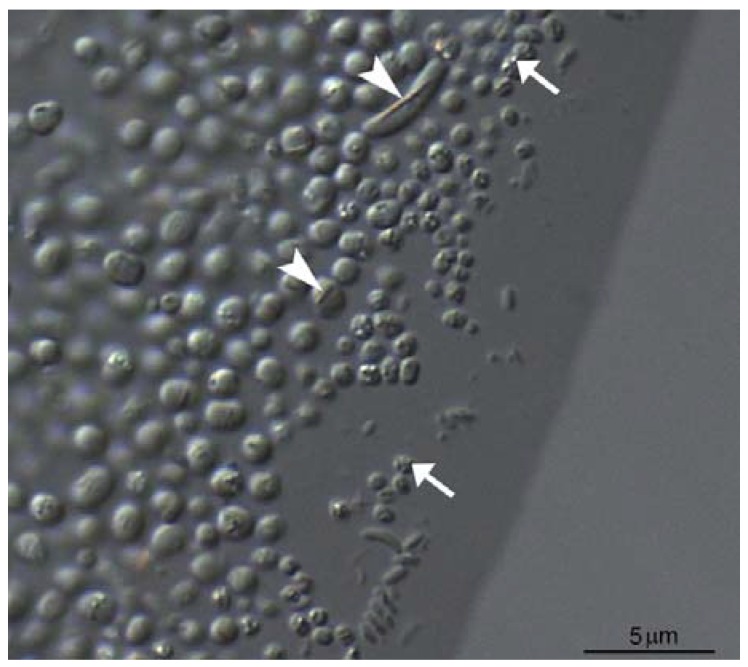
Differential interference contrast (DIC) microscopy image of magnetotactic bacteria (MTB) collected from the Itaipu Lagoon, a brackish lagoon connected to the Atlantic Ocean in Brazil. Cells respond strongly to an applied magnetic field and in very large cells it is possible to observe the chain of magnetosomes (shown at arrowheads). Most cells have a coccoid to bean-shaped morphology and cell inclusions other than magnetosomes are visible in some cells (shown at arrows).

As previously stated, MTB are a diverse group in terms of phylogeny, morphology and physiology, but share the ability to biomineralize magnetosomes usually organized in chains within the cell [1]. Other characteristic features of MTB are a Gram-negative cell wall, motility through the action of flagella, and a negative tactic and growth response to atmospheric concentrations of oxygen [41]. The most abundant morphotype of MTB in natural environments are the magnetotactic cocci (Figure 1). These are often detected in large numbers in chemically-stratified water columns or sediments. Other morphologies of MTB include spirilla, rods, vibrios of various dimensions, and the morphologically conspicuous multicellular aggregates [6].

**Figure 2:**
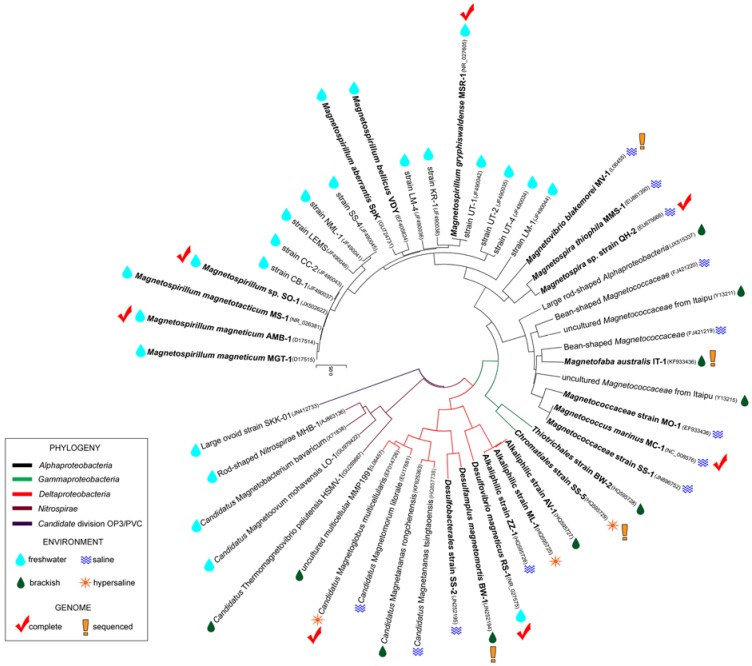
Phylogenetic tree of known MTB based on their 16S rRNA gene sequences. The tree was constructed using the Neighbor Joining method [52] using MEGA software version 5.2 [53]. Taxa in bold type indicate cultured strains, whereas taxa in plain type indicate described but uncultured strains. Accession numbers are given between brackets. Symbols alongside taxa names indicate the environmental type of the strain and the availability of complete or partial genome sequences, as indicated in the accompanying legend.

MTB are generally ubiquitous in aquatic environments [6] and at least once were detected in wet soils [42]. They are usually found in or below the oxic-anoxic transition zone in stratified water columns or sediments [41]. Their occurrence in deep-sea sediments has also been documented [43]. Most cultured MTB were isolated from freshwater or brackish sediments at ambient temperature (these MTB are mesophilic) although their ecological distribution includes saline, hypersaline, polar, thermal, and extreme alkaline habitats [44] (Figure 2). There is evidence that the richness and diversity of MTB in different environments is strongly influenced by salinity [45,46] although temperature, iron availability and concentrations of nitrogen and sulfur compounds are also clearly important in the distribution of MTB [46,47,48,49]. The numbers of MTB cells is any environment is very variable: Their abundance in some marine sediments has been estimated at 10^4^ cells mL^−1^ and 10^6^ cells mL^−1^ in some saline lakes [50].

Metabolically, many MTB are capable of chemolithoautotrophy and chemoorganoheterotrophy and most species are capable of fixing atmospheric nitrogen. All species appear to take up iron very efficiently and due to the synthesis of magnetosomes, MTB may accumulate up to 100 times more iron than other non-magnetotactic heterotrophic bacteria [51]. MTB play significant roles in the biogeochemical cycles not only of iron through magnetosome biomineralization but also of carbon, nitrogen, and sulfur through chemolithoautotrophy.

The best described marine magnetotactic species, the euryhaline *Magnetovibrio blakemorei*, exhibits perhaps the widest metabolic diversity. It is capable of chemolithoautotrophic growth using thiosulfate or sulfide and CO_2_ as electron donors and carbon source, respectively. It also grows chemoorganoautotrophically using formate as electron donor and CO_2_ as carbon source, and chemoorganoheterotrophically using diverse organic acids, amino acids, casamino acids, peptone, yeast extract, and tryptone as sources of electrons and carbon. Autotrophy is through the Calvin-Benson-Bassham cycle [17,54]. It grows under both microaerophilic and anaerobic conditions using oxygen, nitrate or nitrous oxide as terminal electron acceptors. *Mv. blakemorei* is the only known magnetotactic strain capable of growing with both nitrous oxide and nitrate as electron acceptors and fixes nitrogen as well, suggesting that this species plays a significant role in the cycling of nitrogen in marine environments particularly where N_2_O is available as a product of denitrifying bacteria and from ammonia-oxidizing *Archaea* and *Bacteria* [17,55]. The closer phylogenetic relatives to *Mv. blakemorei* are the *Magnetospira* strains. *Magnetospira* *thiophila* strain MMS-1 is also capable of chemolithoautotrophic and chemoorganoheterotrophic growth but uses only oxygen as an electron acceptor [18]. Thiosulfate is the only known electron donor for autotrophic growth and this species has a much narrower range of organic substrates for chemoorganoheterotrophic growth restricted to the organic acids acetate, fumarate, malate, and succinate [18]. Genomic data and growth studies indicate that *Magnetospira* strain QH-2 grows chemoorganoheterotrophically using acetate, citrate, succinate, malate, and fumarate as sources of electrons and carbon but can only respire with O_2_ as a terminal electron acceptor [56]. Although autotrophic growth has not been demonstrated for this species, its genome contains two forms of ribulose-1,5-bisphosphate caboxylase/oxygenase (RubisCO) genes (Forms IAq and II) suggesting that it is able to utilize the Calvin-Benson-Bassham cycle for autotrophy. Alternatively, there is evidence that the reverse tricarboxylic acid (rTCA) cycle might also be used to fix CO_2_ [56]. The genome of *Magnetospira* sp. strain QH-2 contains a number of specific genes that appear to be related to its adaptation to saline environments [56].

All described magnetotactic cocci belong to the *Alphaproteobacteria* class representing a clade that is phylogenetically basal to the *Alphaproteobacteria* [15]. Of this very large group, there are only three cultured strains. All are marine and include: *Magnetococcus marinus* strain MC-1 [15], strain MO-1 [14], and *Magnetofaba australis* strain IT-1 isolated from brackish sediment [16]. While cells of strain MO-1 grow only under chemolithoautotrophic conditions with thiosulfate as electron donor using oxygen as an electron acceptor, *Mf. australis* and *Mc. marinus* display both chemorganoheterotrophic and chemolithoautotrophic growth. Autotrophy in *Mc. marinus* is through the rTCA cycle [57]. *Magnetofaba australis* strain IT-1 represents the first MTB isolated from the Southern Hemisphere and opens new possibilities to study the biomineralization process in strains from different Hemispheres and cell polarities [16].

Other marine strains of MTB are sulfate-reducing bacteria, belong to the *Deltaproteobacteria* class, are anaerobes and produce greigite [20] or greigite and magnetite [24].

### 2.2. Isolation and Cultivation of MTB

The magnetic response of MTB to applied magnetic fields makes them easily detectable in natural samples and facilitates their separation from non-magnetotactic bacteria for further studies. Most descriptions of uncultured strains are based on morphological, phylogenetic, and genomic features determined by culture-independent analyses such as transmission electron microscopy, fluorescent in-situ hybridization and DNA sequencing (using polymerase chain reaction (PCR) with specific primers or single cell genomics) of cells separated from natural samples [10,20,58,59].

Generally, most MTB collected from the Northern Hemisphere (North-seeking, NS) swim parallel to the magnetic field while those from the Southern Hemisphere (South-seeking, SS) swim antiparallel. This unusual characteristic has been exploited to a great degree in many studies in order to harvest MTB from the environment and obtain water samples highly enriched in MTB. The quantity and types of MTB present in natural habitats are strongly dependent on the presence of oxygen concentration gradients and other electron donors such as reduced sulfur compounds (e.g., sulfide) [41]. Increases in the number of specific morphotypes of MTB are often observed in natural samples (microcosms) kept under dim light (to prevent the overgrowth of photosynthetic organisms) and not subjected to mixing, thus stabilizing the oxygen and perhaps other chemical gradients in the sample. Artificial magnetic fields, typically using bar magnets, can be applied to the sample in order to induce MTB to swim in a desired direction for harvesting. Lins *et al.* [60] developed a glass apparatus with two opposite horizontal openings for which NS and SS-MTB are directed to swim when the apparatus, filled with water and sediment, is put inside a magnetic field-inducing coil connected to an energy supply. This electrified coil creates a homogeneous magnetic field in which MTB orient and migrate to the extremity of the openings from where they can be collected. However, this method does not prevent the migration of non-magnetotactic cells to the collection sites due to other tactic responses such as light or oxygen. To prevent this problem, separated cells are often further purified using the magnetic racetrack technique [61] which has now been modified [62]. This technique employs the use of glass Pasteur pipettes that have the thin opening sealed. A cotton plug is placed at the pipette neck and the entire pipette autoclaved. The pipette is filled with filter-sterilized water from the environment up to the cotton plug. Sediment and/or water containing MTB are then added to the wide opening end of the pipette. Magnets are placed close to each end of the pipette to direct MTB towards the closed end of the pipette. After a certain period of time, dependent upon the swimming speed of the MTB under study (typically 20–30 min), the sealed pipette tip can be broken and MTB collected at the extremity can be transferred to culture media or fixatives for microscopy, molecular, and other analyses [41].

Most known MTB appear to be gradient-requiring organisms and grow reasonably well in culture medium with an oxygen concentration gradient and low concentration of nutrients. Such cultures particularly designed for chemolithoautotrophs have been used successfully to isolate new MTB strains since fast growing heterotrophs outcompete fastidious MTB in richer media containing organic carbon sources. Cultured strains of MTB have been isolated using: colony formation, sometimes in shake tubes; repeated rounds of serial dilution to extinction; and magnetic enrichment [41]. Once a specific MTB has been isolated, growth can sometimes be enhanced by using richer heterotrophic media employing different organic and inorganic substrates and various electron acceptors. Growth rates as well as magnetosome production varies greatly even in the same strain depending whether the strain is cultured autotrophically or heterotrophically, aerobically or anaerobically, and with different carbon and iron sources used in the growth medium [29,63,64] (Table 1). Specific culture conditions are thus required for the mass scale production of cells and magnetosomes for biotechnological applications. As far as we know, only three magnetotactic strains are currently being mass cultured to high yields: the freshwater strains *Magnetospirillum magneticum* and *Ms. gryphiswaldense* and the marine vibrio *Magnetovibrio blakemorei*.

**Table 1:** Cultured strains of MTB and their magnetosome characteristics and production under different culture conditions. Numbers between brackets indicate the range of values for each parameter.

Strain	Bacterial Morphology		Magnetosome						References
	Shape	Size (Length × Width) µm	Crystal Shape and Composition	Size (Length × Width) nm	Number/Cell	Size (Length × Width) nm	Number/Cell	Magneto-Some Production	
				Autotrophic		Heterotrophic			
Magnetospirillum magneticum AMB-1	Spirillum	3 × 0.4–0.6	Cuboctahedral magnetite	No growth	No growth	41 ± 15	12 ± 5 (anaerobic)	1.4 × 10^9^ cells mL^−1^; 2.6 mg L^−1^ magnetite (=2.8% cell weight)	[12,29]
						SF = 0.78			
						(anaerobic)			
						33 ± 8.5			
						SF = 0.89	7 ± 4 (aerobic)		
						(aerobic)			
Magnetospirillum magnetotacticum MS-1	Spirillum	4–6 × 0.25	Cuboctahedral magnetite	No growth	No growth	42 (25–55)	17.6 (5–41)	0.2–0.6 g cell L^−1^ (wet weight)	[13,65]
						SF = 0.9			
Magnetospirillum Gryphiswaldense MSR-1	Spirillum	1–20 × 0.7	Cuboctahedral magnetite	NI	NI	Ø 46 ± 6.8 (14–67)	23.4 ± 0.9	41.7 mg L^−1^ (16.7 mg L^−1^ day^−1^)	[66,67]
						SF = 0.91			
Magnetovibrio blakemorei MV-1	Vibrio	1–3 × 0.2–0.4	Elongated prismatic magnetite	48 ± 5 (30–59) × 26 ± 7 (28–40)	17 ± 4 (7–23)	60 × 40	15.34 ± 4	15.14 mg L^−1^; (4.98 mg L^−1^ day^−1^)	[39,68,69]
				AR = 1.8 ± 0.3		SF = 0.65			
Magnetospira thiophila MMS-1	Spirillum	1–3 × 0.2–0.5	Elongated octahedral magnetite	NI	NI	61 ± 12 (22–85) × 52 ± 11 (18–80)	17 ± 5 (8–31)	NI	[18,69]
						AR = 1.2 ± 0.1			
						SF = 0.85			
Magnetospira thiophila QH-2	Spirillum	2.0 ± 0.4 (1–3) × 0.8 ± 0.2	Elongated octahedral magnetite	NI	NI	81 ± 23 × 58 ± 20 SF = 0.71 ± 0.11	16 ± 5 (7–28)	NI	[19]
Magnetofaba australis IT-1	Faba-bean	1.4 ± 0.3 × 1.1 ± 0.3 (*n* = 130)	Elongated octahedral magnetite	NI	6 ± 4 (*n* = 100)	83 ± 26 × 74 ± 23	10 ± 3 (*n* = 100)	NI	[16]
						SF = 0.89 ± 0.05			
Magnetococcus marinus MC-1	Cocci	Ø = 1–2 µm	Elongated pseudo-hexagonal prismatic magnetite	72 ± 11 (33–95) × 70 ± 13 (29–87)	10 ± 2 (6–15)	83 ± 14 (30–110) × 78 ± 11 (15–107)	14 ± 3 (8–19)	NI	[15,69]
				AR=1.2 ± 0.2		AR = 1.2 ± 0.1			
				SF = 0.93					
Magnetococcus MO-1	Ovoid	1.33 ± 0.19 × 1.85 ± 0.40	Elongated cuboctahedral magnetite	No growth	No growth	64 ± 20 × 57 ± 17	17 ± 5	NI	[14]
						SF = 0.89			
Strain BW-2	Rod	4.4 ± 0.6 × 2.2 ± 0.2 (*n* = 62)	Cuboctahedral magnetite	67 ± 16 × 63 ± 15	30 ± 9 (*n* = 46)	No growth	No growth	NI	[25]
				SF= 0.94 ± 0.04 (*n* = 189)					
Strain SS-5	Rod	2.5 ± 0.5 × 1.2 ± 0.1 (*n* = 64)	Elongated prismatic magnetite	86 ± 27 × 63 ± 19	20 ± 7 (*n* = 45)	NI	NI	NI	[25]
				SF = 0.74 ± 0.07 (*n* = 171)					
Desulfovibrio magneticus RS-1	Vibrio	3–5 × 1	Bullet-shaped magnetite	No growth	No growth	Mean length = 60 nm (32–85 nm)	12–15	NI	[23,70]
						SF = 0.5			
Candidatus Desulfamplus magnetomortis BW-1	Rod	**≈** 4 × ≈1	Bullet-shaped magnetite and/or pleomorphic greigite	No growth	No growth	Mean length = 55 nm	NI	NI	[24,71]
						SF = 0.6			

AR = aspect ratio; NI = Not indicated (values are not present in the literature); SF = Shape factor.

### 2.3. Magnetosomes

**Figure 3:**
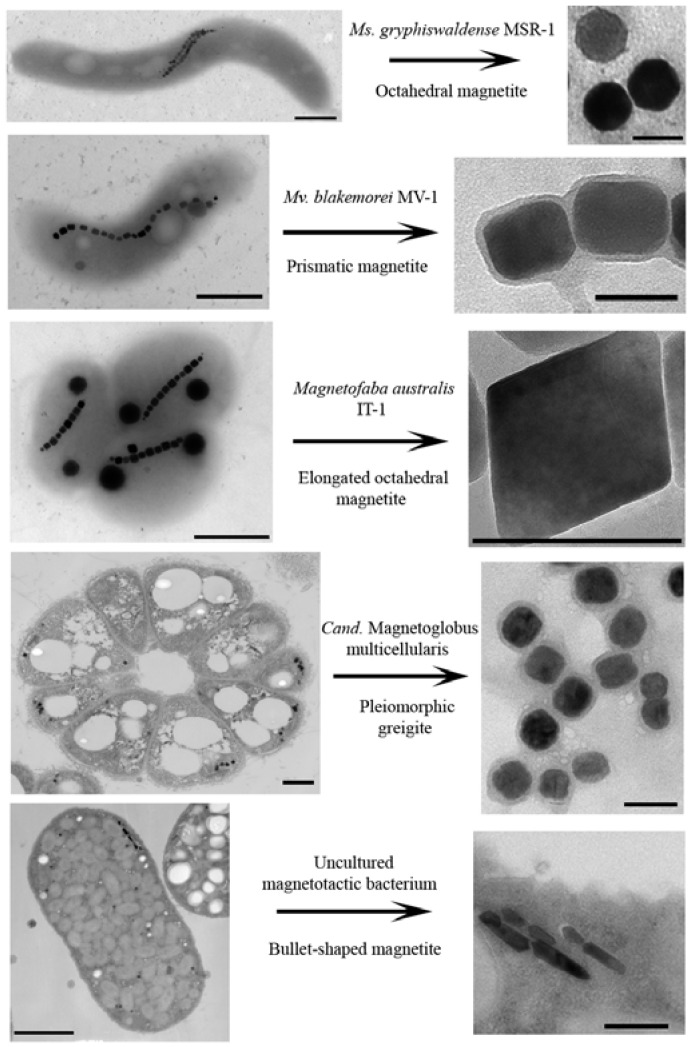
Transmission electron microscopy images of several different MTB showing their distinctive cell and magnetosome crystal compositions and morphologies. Scale bars = 500 nm in bacterial images and 100 nm in magnetosomes images.

Magnetosomes are defined as intracellular organelles composed of magnetic iron mineral crystals individually surrounded by a phospholipid bilayer [27]. The size of the magnetic crystal usually varies from 35 to 120 nm between species. Within this size range, magnetic crystals are a single magnetic domain meaning that they have a permanent magnetic moment at ambient temperature [32]. They are usually organized in one or more chains within the cell, parallel to the axis of motility which, in most cases, is the long axis of the cell. The magnetic moment of the cell, in a magnetic field, imparts a torque to the cell, forcing it to realign along the direction of the applied field. In this way, magnetosomes work as a cellular magnetic compass [62]. The mineral portion of magnetosomes is composed of magnetite (Fe_3_O_4_) or greigite (Fe_3_S_4_) [72]. Both magnetite and greigite crystals share the same general morphologies: cuboctahedral (roughly cuboidal), elongated prismatic (rectangular in projection) or bullet-shape [2,62]. Chains of magnetosomes often lie next to or near the cytoplasmic membrane where they, in some cases, appear to be anchored. The number of chains and of magnetosomes in a chain varies from species to species (Figure 3) and in the same species, it often varies according to environmental conditions [33].

The magnetosome membrane (MM) originates from an invagination of the cell membrane and is likely the first step in magnetosome biomineralization [73]. Different proteins are involved in this step and their recruitment changes the protein composition of the MM [27]. The following steps, that apparently occur simultaneously, are iron uptake, crystal nucleation, crystal maturation, and the alignment of magnetosomes into chains [74]. Iron is precipitated inside of the nascent vesicles to form magnetic crystals and, during the maturation of magnetosomes, most of these invaginations detach from the cell membrane and appear to become true vesicles [75].

**Figure 4:**
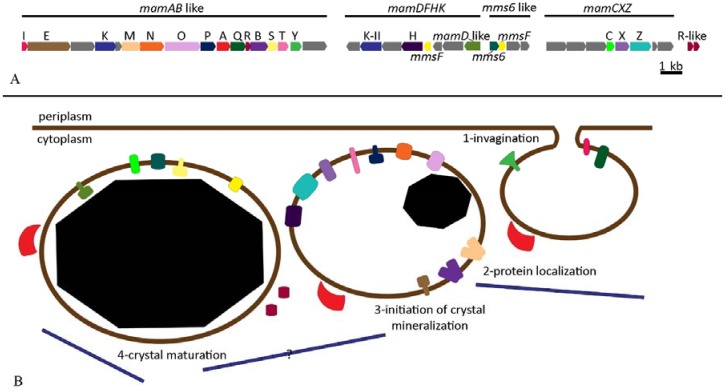
Proposed mechanism of magnetosome biomineralization in *Magnetovibrio blakemorei* strain MV-1. The putative magnetosome island (MAI) of *Mv. blakemorei* (A) [5] and the putative function of each encoded protein during magnetosome formation (B) based on their similarities to proteins described for *Magnetospirillum* species. The color of each ORF is used to identify the localization of encoded proteins. Unidentified genes in grey encode hypothetical proteins. The *mamL*, *J*, *U*, *G*, and *F* genes, although present in *Magnetospirillum* species, are not present in the MAI of *Mv. blakemorei*.

Magnetosome biomineralization is a genetically controlled process that involves approximately 28 proteins encoded by the so-called *mam* and *mms* genes. In *Magnetospirillum gryphiswaldense*, these genes are organized in four operons: the *mamAB*, *mamGFDC*, *mamXY* and *mms6* operons [5,76]. In *Ms. gryphiswaldense*, only the *mamAB* operon is essential for magnetosome synthesis while the absence of the other operons does not lead to the absence of magnetosomes but to differences in magnetite crystal morphology and the production of particles not clearly organized in chains [77]. The operons, localized in a larger cluster in the genome of *Ms. gryphiswaldense* and of some other MTB, represent a genomic island referred to as the MAI standing for Magnetosome Island [76]. This genomic region contains genes responsible for iron transport, magnetite crystal nucleation and growth, magnetite crystal morphology, and magnetosome organization within the cell (Figure 4). Comparative studies based on cultured and uncultured magnetite- and greigite-producing MTB show that the *mamABEIKMOPQ* genes are strongly conserved among different species [73,74,75,76,77]. Although the function of many of these genes has not yet been elucidated, they have been inferred from similarities to other known proteins. One of the more conserved proteins is MamK, a homolog of the prokaryotic cytoskeleton protein MreB [78]. MamK is an actin-like protein that forms interconnected filaments along the cell. Magnetosomes are linked to this long structure by another protein called MamJ, although the gene coding for this protein is absent from the genomes of many MTB including *Magnetovibrio blakemorei*, *Magnetofaba australis*, and *Magnetococcus marinus*. In these bacteria, a hypothetical protein is encoded by a gene adjacent to *mamK* but it is not clear whether this protein functions similarly to MamJ. The fixed organization of magnetosomes into chains and its connection to the cell membrane enable the cells to orient along magnetic field lines since the torque exerted by magnetosomes chain is transferred to the whole cell [41].

Other proteins encoded by genes in the *mamAB* operon are thought to be related to the invagination process (*mamB*, *E*, *I*, *L*, and *Q*), to iron transport (*mamB* and *M*), and to magnetite biomineralization (*mamE*, *O*, *T*, *P*, and *S*) [73,79,80,81]. The *mamXY* operon encodes proteins related to the magnetosome membrane (*mamY*, *X*, *Z*, and *ftsZ*-like genes) and its deletion causes cells of *Magnetospirillum* to produce smaller magnetite particles with superparamagnetic characteristics [77,82]. Crystal size and shape are mainly regulated by proteins encoded in the *mamCD* operon (composed of the genes *mamC*, *D*, *F*, and *G*) and its deletion also leads to a reduction of the size of the magnetite magnetosome crystals [83]. The *mms6* operon contains five genes (*mms6*, *mmsF*, *mgr4070*, *mgr4071*, and *mgr4074*) [74] that also appear to be involved in magnetite crystal shape and size. The deletion of both the *mamCD* and *mms6* operons seriously affects both the morphology of the magnetite magnetosome crystals and the alignment of the magnetosomes [83]. Recently, the entire set of magnetosome genes from *Magnetospirillum gryphiswaldense* were genetically introduced into *Rhodospirillum rubrum*, a non-magnetotactic photosynthetic bacterium causing it to express biomineralization of functional magnetite magnetosomes structurally similar to those produced by *Magnetospirillum* and which conferred a magnetic moment to the host cell [40].

Studies involving specific functions of magnetosome proteins (Table 2) have enabled the use of synthetic peptides that mimic the function of these proteins in the chemical synthesis of magnetite nanoparticles resulting in the synthesis of magnetite crystals with some of the desirable characteristics of magnetome crystals [84]. Knowing the localization of specific magnetosome proteins in the MM and their pattern of expression provide the opportunity to choose the best target for protein modifications, for example, gene fusions between a *mam* gene and a gene encoding a protein of catalytic interest [85]. Investigating proteins directly related to magnetosome crystal size and morphology remains an important but partially unexplored direction of research but being able to control these crystal characteristics will likely result in different physical and magnetic attributes for magnetosome specific applications. An excellent example of this is in hyperthermia treatment of tumors in which heat, generated by magnetite magnetosomes, even when the crystals have oxidized to the less magnetic mineral maghemite, subjected to an alternating magnetic field, is used to kill tumor cells. The amount of heat generated is dependent on magnetosome crystal size and morphology, and the distribution of particles inside the tumor further affects the efficacy of this treatment [86,87]. It has also been demonstrated that the size of magnetosome magnetite crystals affects their use as contrast agents in magnetic resonance imaging (MRI) [88].

**Table 2:** Specific magnetosome proteins with their respective cell localization and putative functions in magnetite biomineralization by MTB.

Protein	Localization	Process	Function	Deletion Effects	References
MamA	Cytosol. Dynamic, surrounding vesicles	Invagination of cell membrane	It has multiple domains with TPR motifs (protein-protein interactions); may act as multi-protein assembly site; stabilizes magnetosome chain.	Invagination is not affected. Reduction in the number of magnetosomes and changes in iron accumulation.	[89,90,91]
MamB	Transmembrane in MM	Iron transport and magnetite nucleation	May be involved in iron transport since has homology to CDF (cation diffusion facilitator). Contains TPR domain (protein-protein interactions) and interacts with MamE; requires MamM for stabilization.	Loss of magnetosome vesicles and of crystal formation.	[74,79,89]
MamC	Transmembrane in MM	Crystal shape and size	Its loop interacts with magnetosome crystal. It is not essential to biomineralization but may control chemical conditions inside vesicles.	Changes in size and organization of chains and size of vesicles. No effects observed in crystal size or shape.	[78,89]
MamD	Transmembrane in MM, *N*-terminal in ML	Crystal shape and size	Associated with control over size of magnetosome crystal.	Changes in crystal size.	[78,89]
MamE	Transmembrane in MM, *C*-terminal towards ML	Iron transport and nucleation	Acts as a serine protease and has PDZ domain (protein-protein interaction) which interacts with MamB and I. Magnetochrome might control the magnetosome redox state and balance between Fe^2+^/Fe^3+^.	Formation of empty magnetosome vesicles, loss of magnetite synthesis, mislocation of MamI and other Mam proteins.	[74,80,89]
MamF	Transmembrane MM	Crystal shape and size	Associated to control of magnetosome size; interacts with crystal.	Changes in crystal size.	[78,89]
MamG	Transmembrane in MM	Crystal shape and size	Associated to control of magnetosome size.	Changes in crystal size.	[78,89]
MamH	Transmembrane in MM	Iron transport and nucleation	Contains conserved domains homologous to MFS proteins (membrane transporters) and might function as phosphate transporter during magnetite biomineralization.	Reduced number and size of magnetosomes.	[74,89,92,93]
MamI	Transmembrane in MM	Invagination	Involved in the formation and bending of the MM.	Absence of MM.	[28,74,89]
MamJ	Cytosol	Arrangement of chains	Acts as an anchor between MamK filaments and vesicle membrane to arrange magnetosomes in a chain.	Magnetosomes arranged in clusters and no longer in chains. Reduced magnetotactic response.	[78]
MamK	Cytosol	Arrangement of chains	Controls chain assembly and position along the cell axis; positions chain for cellular division; homologous to MreB (actin-like).	Lack of filaments near the magnetosomes. Shorter chains and wrong position of MamJ.	[94]
MamL	Transmembrane in MM	Invagination	Involved in the formation of MM; similar to MamI.	Absence of MM.	[74]
MamM	Transmembrane in MM	Iron transport and magnetite nucleation	Involved in iron transport and may use H^+^/cation antiporter mechanism. Involved in the begining of crystalization and localization of other Mam proteins; stabilizes MamB; homologous to CDF (cation diffusion facilitator).	Loss of magnetite crystals, formation of empty vesicles.	[74,79,89]
MamN	Transmembrane in MM	Iron transport and magnetite nucleation	Homologous to Na^+^/H^+^ antiporter and might be involved in the extrusion of H^+^ from the vesicle.	Formation of empty magnetosome vesicles. Does not affect localization of other proteins.	[28,74,89]
MamO	Transmembrane in MM; *C*-terminal in ML	Iron transport and magnetite nucleation	Composed of two domains: (1) transmembrane, homologous to proteins involved in transport of anions across cell membrane and (2) similar to a trypsin-like peptidase, but possibly with no protease function.	Formation of empty magnetosome vesicles.	[74,89]
MamP	Transmembrane in MM with active sites towards ML	Iron transport and magnetite nucleation	Involved in control of crystal number and size and in electron transfer necessary to magnetosome assembly and magnetite formation; similar to MamE and MamT; may contain an iron-binding site.	Defects in crystal size, fewer magnetosomes per cell.	[74,89,95]
MamQ	Transmembrane in MM; *C*-terminal in ML	Invagination	Unknown function; homologous to LemA.	Complete loss of magnetosome formation in AMB-1.	[74,89]
MamR	Cytosol	Crystal shape and size	Controls the number and size of crystals; predicted to have a DNA-binding domain.	Smaller magnetosome and weaker magnetotactic response.	[74,80,89]
MamS	Transmembrane in MM; *C*-terminal in ML	Crystal shape and size	Controls the number and size of crystals.	Defects in crystal size and morphology, weaker magnetotactic response.	[74]
MamT	Transmembrane in MM; *C*-terminal in ML	Iron transport and magnetite nucleation	Involved in regulation of crystal size and morphology; has a magnetochrome domain.	Defects in crystal maturation and loss of magnetotactic response.	[74,89,95]
MamU	Cytosol	Invagination	Unknown function. Homologous to DGK Family, that includes kinase involved in regulation of cell response.	None observed.	[74,89]
MamV	Transmembrane in MM	Iron transport and magnetite nucleation	Putative CDF transporter.	None observed.	[74,79]
MamW	MM (structure unknown)	Iron transport and magnetite nucleation	Implicated it magnetite synthesis or not associated to magnetosomes.	None observed.	[76,77]
MamX	Transmembrane in MM; *C*-terminal in ML	Iron transport and magnetite nucleation	Involved in electron transport, with Cytochrome c-like domain; weak similarity to MamS and E.	Smaller crystals and with irregular shapes. Weaker magnetotactic cell response.	[89,92]
MamY	Transmembrane in MM; *C*-terminal in cytosol	Invagination	Constricts the MM and consequently affects crystal growth; homologous to BAR proteins (involved in membrane dynamics).	Enlarged magnetosome vesicles with smaller crystals.	[96]
MamZ	Transmembrane in MM; *C*-terminal in ML	Iron transport and magnetite nucleation	Involved in redox control for magnetosome formation; creates an iron oxidoreductase and transport complex with MamX and MamH.	Smaller size of crystals and higher proportion of twinned crystals.	[89,92]
Mms6	Transmembrane in MM	Crystal shape and size	Involved in the initiation of magnetite synthesis and control of crystal shape; presents *in vitro* activity.	Smaller magnetosomes with heterogeneous shapes. Irregular alignment of chains.	[89,97]
MmsF	Transmembrane in MM	Crystal shape and size	Involved in the control of size and shape of magnetite crystal during maturation.	Formation of elongated crystals and of non-magnetotactic cells.	[74,89]

BAR—Bin/Amphiphysin/Rvs domain related to membrane dynamics; CDF—cation diffusion facilitator; DGK—diacylglycerol kinases family; MM—magnetosome membrane; ML—magnetosome lumen; PDZ—conserved domain related to protein-protein interaction; MFS—major facilitator superfamily of secondary transporters; TPR—Tetratricopeptide repeat domain related to protein-protein interactions.

### 2.4. Mass Production of MTB and Magnetosomes

Most studies aimed at large-scale production of magnetosomes have involved *Magnetospirillum* species and, therefore, most of the available information on magnetosome synthesis is based on strains of this genus. *Magnetospirillum* species produce cuboctahedral magnetite crystals about 40–45 nm in diameter. Each cell can possess up to approximately 60 magnetosomes organized as a single chain [98] although the actual number of magnetosomes per cell is clearly dependent on culture conditions. *Magnetospirillum* species are chemoorganoheterotrophic and use organic acids as source of carbon and electrons although autotrophy based on the oxidation of reduced sulfur compounds has been demonstrated in some strains [67]. Generally members of this genus grow microaerophilically or anaerobically, utilizing O_2_ and nitrate as electron acceptors, respectively, although one species, *Ms. magnetotacticum*, appears to require O_2_ even when respiring with nitrate [99]. Despite the fact that the magnetite oxygen originates from water, O_2_ concentration, as well as the presence of nitrogen oxides, are important factors that directly affect magnetite biomineralization in this genus [100]. Microaerobic conditions are required for growth and production of magnetite in magnetosomes of *Ms. magnetotacticum* [38]. Despite the fact that cells grow in sealed flasks containing 0.1% to 21% oxygen in the headspace, concentrations higher than 5% inhibit magnetite magnetosome synthesis [31]. Iron uptake is also stimulated under microaerobic conditions [99]. The role of O_2_ in magnetite biomineralization is not clear, but the most accepted explanation is that it is required to establish optimal redox conditions for synthesis of magnetosomes and growth [101]. Moreover, *Magnetospirillum* species are relatively easy to grow especially compared to marine species of MTB, and have tractable genetic systems [40,99,102].

However, the recently described *Magnetovibrio blakemorei* utilizes a wider range of metabolic substrates, can grow and produce magnetosomes using a wider range of electron acceptors [17] and is amenable to mass cultivation in large scale [39]. Thus, this species is an excellent candidate for the mass production of magnetosomes. Moreover, cells of *Mv. blakemorei* produce elongated prismatic crystals of magnetite, a characteristic that results in a particle with a stronger magnetic anisotropy thereby facilitating their manipulation by external magnetic fields [87]. These magnetosome crystals also have a larger surface-to-volume ratio than the cuboctahedral crystals of *Magnetospirillum* [103], a characteristic of special interest for applications involving adhesion or the expression of proteins in the MM, since it provides a larger surface area for substrate binding. Most applications require that magnetic nanoparticles have a controllable, consistent magnetic anisotropy and, therefore, that they have a defined and preferentially elongated shape [87]. It has been demonstrated that magnetosome vesicles in *Mv. blakemorei* are elongated prior to magnetite crystal formation suggesting that they predefine crystal morphology at least in this species [103]. Although the gene for MamK filaments was detected in the genome of *Mv. blakemorei*, cryomicroscopy images of frozen cells revealed only fragmented filaments that were not attached to magnetosome vesicles or to the poles of the cell (Figure 4). Moreover, magnetosome vesicles directly connected to the cell membrane were not found suggesting that they detach shortly after invagination [103]. These features indicate that magnetosome formation in *Mv. blakemorei* differs from that described for *Magnetospirillum* species although it is clear that genetic studies are required to clarify this process. Such studies are currently difficult to perform because of a lack of a reproducible genetic system in *Mv. blakemorei*.

To obtain high yields of magnetite magnetosomes from *Magnetospirillum* species, cells are generally grown in a bioreactor chemoorganoheterotrophically with O_2_ as the terminal electron acceptor, succinate or lactate as the electron and carbon source, and nitrate or ammonium ions as the nitrogen source [38,104,105]. Since magnetosome formation is higher under microaerobic conditions [101], the main drawback to mass culture is the need to maintain strict control over the dissolved O_2_ concentration in the growth medium. There are a number of published studies, most using *Magnetospirillum* species as the model MTB, involving comparisons of growth media and the optimization of growth conditions for maximum magnetosome yields by MTB. Growth and magnetosome production was compared between *Ms. magneticum*, *Ms. magnetotacticum* and *Ms. gryphiswaldense* all grown in a 5 L bioreactor under the same conditions [38]. *Ms. gryphiswaldense* had the highest growth rate and showed the highest tolerance to O_2_ [38]. Although growth of *Ms. gryphiswaldense* was not impaired by variations in O_2_ concentration, iron uptake and the cellular magnetotactic response was reduced by half with increasing O_2_ concentrations [38]. The maximum magnetosome yield was of 6.3 mg magnetite L^−1^·day^−1^ by *Ms. gryphiswaldense* and 3.3 mg and 2.0 mg for *Ms. magneticum* and *Ms. magnetotacticum*, respectively, when these cells were cultured under a constant O_2_ tension of 0.25 mbar and a growth medium containing lactate as the carbon source and ferric citrate as the iron source (Table 3) [38]. Although the methods used to estimate magnetosome productivity varies greatly, the values presented here are comparable to those achieved in cultures of *Magnetovibrio blakemorei*: 4.98 mg magnetite L^−1^ day^−1^ prior to optimization of the growth medium [39]. The utilization of iron-chelating agents such as hemoglobin (0.4 µM) and EDTA (ethylenediamine tetraacetic acid at 4 µM) in the growth medium resulted in up to a 6-fold increase in crystal production by *Ms. magneticum* cells. Moreover, the chains produced in the presence of iron-chelating agents were longer and with improved heating capacities when subjected to an alternating magnetic field [106]. These experiments were carried out in 10 or 500 mL flasks and a rationale of cost to benefit is necessary for the utilization of chelating agents in large-scale cultures.

**Table 3:** Magnetite production by MTB in large scale mass cultures.

MTB	Culture	Medium	Magnetite production (mg L^−1^) *	Magnetite productivity (mg L^−1^ day^−1^) *	References
*Ms. magneticum*	Fed-Batch	MSGM	9 ± 0.7	3.7 ± 0.13	[104]
*Ms. gryphiswaldense*	Batch	LSM	7.9	6.3	[38]
*Ms. gryphiswaldense* NPHB	Fed-Batch	OFM	58.4 ± 6.4	-	[107]
*Ms. gryphiswaldense*	Fed-Batch	OFM	41.7	16.7	[105]
*Ms. gryphiswaldense*	Fed-Batch	OFM	83.23 ± 5.36	55.49	[108]
*Ms. gryphiswaldense*	Fed-Batch	OFM	356.52	178.26	[109]
*Ms. gryphiswaldense*	Semi-continuous	OFM	168.3	83.5	[109]
*Mv. blakemorei*	Batch-flask	[17]	15.14	4.98	[39]
*Mv. blakemorei*	Batch-flask	Optimized	64.35	16.09	[39]
*Mv. blakemorei*	Batch	Optimized	22.4	5.6	[39]
*Mv. blakemorei*	Fed-Batch	Optimized	26	3.2	[39]

MSGM: Magnetic Spirillum Growth Medium [13]; LSM: Large Scale Medium [35]; OFM: Optmized Flask Medium [94]. * Estimates of magnetite production and their error bars (when present) are given as reported in reference articles.

*Magnetospirillum gryphiswaldense* was cultured in a 42 L fed-batch bioreactor in which the concentrations of lactate as carbon source and O_2_ were rigorously controlled [108]. The strategy was to stimulate growth by initially increasing the O_2_ concentration to a relatively high level and then allowing bacterial respiration to reduce partial pressure of O_2_ to levels optimal for magnetosome magnetite synthesis and repeating this cycle by introducing more O_2_ and increasing the speed of stirring [108]. This methodology was used to satisfy the culture’s different requirements for O_2_ for growth and also for magnetosome production while later maintaining the dissolved O_2_ concentration at a threshold for both. This resulted in a magnetite yield of 16.7 mg magnetite L^−1^ day^−1^. Magnetosome production by *Ms. gryphiswaldense* increased with further adjustments of the stirring rate and air flow to the culture to control the dissolved O_2_ at an optimal level for magnetite synthesis and the pH-stat feeding of nutrients to maintain the concentrations of ferric citrate and lactate between 70–110 mM and 3–6 mM, respectively (Table 3) [108].

Modifications to the growth medium and incubation conditions have been used by many different research groups with the goal of maximizing growth and magnetosome production at minimum expense. For example, the constant input of sodium lactate and NH_4_Cl in the fed-batch strategy resulted in the accumulation of Na^+^ and Cl^−^ ions which increased the osmotic potential of the medium negatively affecting the growth of *Ms. gryphiswaldense* [109]. The substitution of carbon and nitrogen sources for lactate and NH_3_, respectively, in the feed solution led to an increase in cell growth and magnetosome production in a bioreactor of 42 L [109]. Growth and magnetosome production can also be increased by employing a semi-continuous culture strategy. In a 7.5 L bioreactor, the first stage of semi-continuous culture of *Ms. gryphiswaldense* was maintained until late exponential growth (40 h) with a magnetosome production of 168.29 mg L^−1^ day^−1^ and then 10% of the volume of this culture was used to inoculate the second stage of the culture which reached a magnetosome yield of 83.54 mg L^−1^ day^−1^ after 28 h (Table 3) [109].

Optimization of the growth medium of *Magnetovibrio blakemorei* led to a magnetosome yield of 22.4 mg L^−1^ after 96 h when cells were grown in a 2 L bioreactor. Further pulses of iron injected in the bioreactor increased magnetosome production to 26 mg L^−1^ although magnetosome synthesis then decreased after relatively long incubation times (e.g., 196 h) [39] (Table 3).

Differences observed in magnetosome production and magnetite productivity by different MTB (Table 3) are certainly due to the different strain characteristics and culture conditions. However, the methodologies utilized to estimate magnetite production by each strain is often difficult to compare directly in these studies. Measurements of magnetite production have been determined by counting the number of cells and the number and size of magnetosomes per cell, thereby allowing for the calculation of the number of magnetosomes per mL [39]. Estimates based on extracted magnetosomes using spectrophotometry [107] have also been reported but in many studies the way magnetosome production is estimated is not clear.

**Figure 5:**
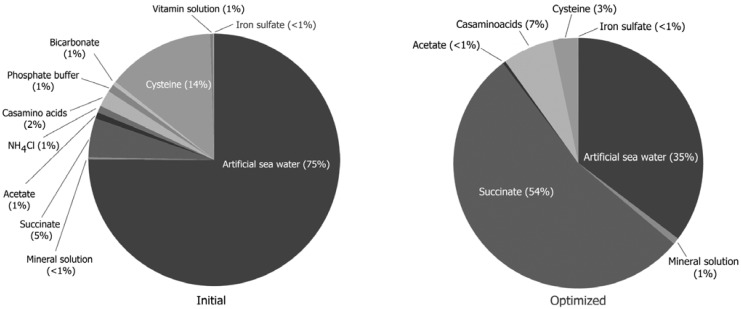
Comparative analysis of the cost of each growth medium component as a percentage of the initial and optimized media for *Magnetovibrio blakemorei*. Medium components have been described previously [36].

Optimization of the growth medium for *Magnetovibrio blakemorei* was recently achieved using a statistics-based experimental design which involved the removal of some components and an increase in the concentration of others [39]. In this study, we evaluated the relationship between the cost of the initial and optimized culture media in relation to culture productivity (cell yield) and estimated the cost of each medium component before and after optimization (Figure 5). The increased amount of sodium succinate as the carbon source raised the cost substantially while the elimination of certain components, such as the vitamin solution, reduced the cost of the medium and was pivotal to balancing the cost and production in this case. Nevertheless, optimization of the growth medium resulted in an increase in the overall cost by a factor of 2 (from $2.50 to $5.04 USD per L) while magnetosome production increased by 8-fold, resulting in a four-fold net increased production.

Besides modifications in growth media and culture conditions, magnetosome productivity can be modified through genetic manipulation of the magnetotactic strain used. For example, a mutant strain of *Magnetospirillum gryphiswaldense* with a higher O_2_ consumption rate during growth and a lower accumulation of intracellular granules of poly-ß-hydroxybutyrate (PHB) produced more magnetosomes than the wild-type strain under the same culture conditions [107]. On the other hand, magnetosome production decreased in cultures of a mutant of this same organism that produces higher amounts of PHB granules (compared to the wild-type) grown in a bioreactor [107]. Genetic engineering of MTB is also of premier importance when the goal is to modify the expression of specific magnetosome proteins in the MM. This strategy of modifying the MM through genetics offers advantages in comparison to chemical functionalization, such as the correct positioning of the expressed proteins where the catalytic site (if present) is exposed to the appropriate substrates thereby preserving the catalytic activity of purified enzymes. This is significant since chemical immobilization procedures involving proteins could lead to loss of enzyme activity [85].

**Figure 6:**
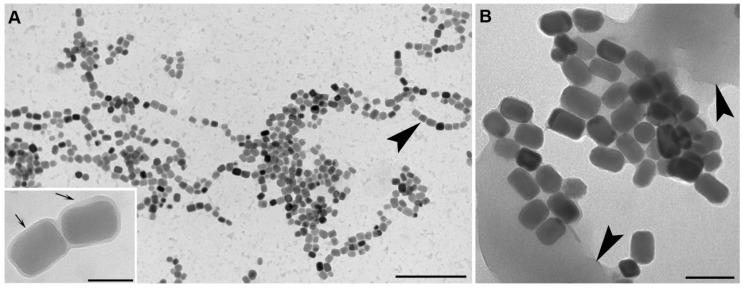
Magnetosomes purified from cells of *Magnetovibrio blakemorei* strain MV-1. Magnetosomes purified from cells lysed using physical methods or alkaline lysis (**A**) with the magnetosome membrane (MM) shown in the inset (at arrows). Note that after this treatment most magnetosomes remain in chains (at arrowhead in A); Some physical-chemical methods lead to magnetosomes losing their membranes and arrangement, forming clumps due to magnetic interactions between magnetosome crystals (**B**). Cell debris (arrowheads in B) is generally always present in poorly washed suspensions of magnetosomes reducing purity of the preparation and potentially interfering with specific applications of the isolated magnetosomes. Scale bars = 1 μm in A (100 nm in inset), 150 nm in B.

After magnetosome production, it is necessary to separate and purify magnetosomes or magnetosome crystals for use in the majority of biotechnological applications. Magnetosomes have been successfully purified from cells of MTB using a number of different procedures. Harvested cells of MTB must be first lysed prior to magnetosome purification. After cell lysis, magnetosomes can be separated from cell debris and non-lysed cells by exploiting their magnetic properties using relatively strong magnets. Cell disruption can be achieved by ultrasonication, alkaline lysis, and by use of a French press or a high-pressure homogenizer [107,108,109]. Importantly, the MM lipid bilayer is maintained as a coherent structure around the magnetite crystals with all these techniques [107,108,109]. Removal of the lipid membrane is possible with the use of detergents such as sodium dodecyl sulfate (SDS), allowing for the purification of the magnetosome magnetite crystals which tend to agglomerate due to the magnetotactic interactions between particles after detergent treatment [110]. Extensive washing of magnetosome or magnetosome crystals after separation is crucial to obtain clean material suitable for further use since cell debris (e.g., membranes) including electrostatically-charged cell proteins that might associate with the MM but are not part of it, are difficult to remove and could interfere with the performance of magnetosomes in specific applications (Figure 6).

Advances made in the mass culture of MTB and the mass production of magnetosomes and the general need for large amounts of material for specific applications has led to the development of continuous magnetosome purification procedures at large scale. These techniques involve the lysis of large quantities of cells, which are disrupted using a high-pressure homogenizer, followed by separation of magnetosomes from cell debris by passing the lysate through a magnetic column composed of a material that is strongly magnetic when placed in a very high strength magnetic field. Magnetosomes remain in the column and can be washed repeatedly as other materials pass through the column. Magnetosomes are recovered from the column by removing the external magnetic field and passing buffer or water to wash out the magnetosomes. Further treatment of magnetosomes to remove surface proteins and cell DNA is carried through low-power sonication and the use of urea and proteinase K [111]. This process is important for *in vivo* applications of nanosized magnetite particles, such as drug delivery, where strict standards of purification are required to avoid toxicity and immunological responses to the MM and extraneous proteins [112]. For biotechnological applications where it is necessary to preserve the MM and the surface proteins of functionalized magnetosomes, treatment of the magnetosomes with urea and proteinase K can be eliminated. Alternatively cells could be lysed using a French pressure cell for cell disruption followed by magnetosome purification as described above. The use of either procedure preserves the activity of magnetosomes functionalized with luciferase-MagA [113], other GFP-fusion proteins, and enzymes [85,114]. These processes can be used to isolate non-modified magnetosomes that can be lyophilized and/or sterilized by γ-rays and stored for further use. These isolated magnetosomes have low toxicity to different kinds of mammalian and human cells including H22, HL60, EMT-6 cells or mouse fibroblasts, and to live mice [112,115].

### 2.5. Biotechnological Applications of Magnetosomes

Nanometer-sized magnetic particles are of great interest in biotechnology since they have a large surface area which can be used for anchoring relatively large amounts of specific molecules and can be easily manipulated using an external magnetic field. These magnetic particles including magnetosomes can be bound to proteins, cells, viruses, or genes of interest which can be then be subsequently separated using magnetic techniques [116]. The particles most often used for these types of studies consist of iron oxides especially magnetite and maghemite (γ-Fe_2_O_3_) which are more stable than iron sulfides such as greigite. They have been used in various biomedical applications such as immunoassays, cell separation, hyperthermia protocols (treatment of cancer by localized heating), drug carriers, nuclear magnetic resonance, and others [117]. These applications require that the magnetic particles have high magnetization, consistent sizes smaller than 100 nm, consistent morphologies, and are biocompatible (are non-toxic). Nanoparticles of magnetite can be synthesized abiotically using various processes such as co-precipitation [118], microemulsion [119], electrochemical synthesis [120], hydrothermal synthesis [121], oxidation-precipitation [122], and others. However, these chemically-produced magnetites generally do not have all the desirable features necessary for certain applications but are characteristic of bacterial magnetosomes.

Magnetosome magnetite crystals have high magnetization, consistent species-specific nanometer sizes and morphologies, and based on a number of recent studies, appear to be biological compatible [41]. Their narrow, single magnetic domain size distribution is difficult to achieve using chemical synthesis techniques [87]. This characteristic, their excellent degree of crystallinity, and anisotropy are advantageous particularly in applications in which thermal stability and hysteretic heating are required such as in hyperthermia treatments [87]. Specifically in hyperthermia, it has been shown that magnetosomes are advantageous in relation to synthetic nanoparticles because their organization in chains favors their internalization by cells and permits magnetosomes to be homogeneously distributed inside the tumor, a factor that increases the effectiveness of the treatment in killing tumor cells [123]. In addition, because of the MM, magnetosomes can be functionalized either chemically, which involves chemically attaching specific proteins (e.g., antibodies) or other molecules, or by genetic engineering in which genes encoding proteins of interest are fused to specific magnetosome genes encoding MM protein components [85]. How this coupling is made is important because it can affect the stability and quantity of these molecules bound to each magnetosome and thus directly affect efficacy of the modified magnetosome in specific applications [124]. The amine groups located in the MM have been used for the immobilization of functional molecules. Different molecules can be bound to the amine groups on the MM and further, antibodies can be bound to these molecules [125]. Glutaraldehyde has been successfully used to directly link an antibody to magnetosomes [126] while several antimicrobial peptides were tested for their spontaneous integration into the MM [127].

The surface of the magnetosomes can be modified for use in DNA extraction procedures. These modifications include organic compounds which create an amine layer forming a cover with a positive ionic charge that facilitates the interaction between DNA molecules and magnetosomes, leading to a much higher efficiency in the recovery of DNA than other available magnetic particles kits [128]. Several techniques were developed to express and efficiently display functional molecules attached to the MM. Some MM proteins have their catalytic sites exposed. Genes encoding these MM proteins can be fused to genes encoding proteins of interest, resulting in the correct positioning of the catalytic site on the surface of magnetosomes. Ideally, the anchor protein should be highly expressed to increase the amount of the exposed catalytic site. An excellent example of a MM anchor protein appears to be the MamC protein of *Magnetospirillum magneticum* which is highly expressed in MM and has been used effectively as an anchor display different enzyme complexes [85,114,129]. Other MM proteins including MagA and Mms6 have been used to anchor protein A, luciferase, and an estrogen receptor [130,131].

Mass scale production of modified magnetosomes was assayed in a 10 L bioreactor under microaerophilic conditions with pH-stat feeding of carbon and nitrogen sources. *Ms. magneticum* carrying a plasmid with a fused protein Mag-luciferase was grown and the production of modified magnetosomes was enhanced with addition of cysteine, yeast extract, peptone and ferrous sulfate as iron source. The plasmid inserted for production of the fused protein was stable under these non-selective conditions [130].

The model of magnetosome synthesis has been utilized in producing chemically-synthesized, bioinspired magnetic nanoparticles by precipitating magnetite chemically in the presence of various purified magnetosome proteins including mms6 [84]; this strategy helps to overcome some costs associated with the growth of MTB and the purification of magnetosome crystals for specific applications [84]. Numerous applications of nanosized magnetic particles have recently been reviewed [58,63]. Again, given the high costs involved in the culturing of MTB and magnetosome purification, in large scale for the production of magnetosomes, it seems likely that magnetosomes will only be useful in situations where it makes economic sense such as in biomedical procedures where the high purity of the crystals, low toxicity, and high biocompatibility may be absolutely necessary.

Living and motile MTB have proven useful in bioremediation, cell separation, as carriers of specific molecules, and in the detection of magnetic domains in hard materials. The detection of magnetic fields emitted by different materials, such as rocks and meteorites, can be identified nondestructively with the use of living MTB, since cells swim towards magnetic poles along the direction of the magnetic field [132]. Living MTB interact with different molecules by taking them up within the cell or binding them on the cell wall surface. In this way, they have been shown to have potential in the bioremediation and removal of plutonium radionucleotides and heavy metals [133]. A system was created to allow the interaction of MTB with a solution contaminated with plutonium and subsequently the cells were separated by magnetic orientation thereby recovering the plutonium [134]. *Desulfovibrio magneticus* was used in the recovery of cadmium present in the growth medium [135]. Cells of *Magnetospirillum gryphiswaldense* reduced gold ions to gold nanoparticles that became attached to the bacterial surface and could then be separated using magnetic techniques [136]. Viable granulocytes and monocytes after phagocytizing living cells of MTB can be magnetically purified from blood [137]. Finally, although it is unlikely that regulatory agencies would approve treatments involving the application of living bacteria in the blood stream, it has been shown that MTB have great potential for acting as drugs carriers in the human body. Cells of *Magnetococcus marinus* can carry fluorescent microbeads of 2 µm and swim at their normal speed along magnetic field lines [138]. Cells might therefore be able to efficiently navigate under simulated conditions imitating the interior of the human body. Theoretically, they could be targeted to a specific part of the human body, carrying various types of molecules/drugs and are small enough not to interfere with blood flow. Finally, cells of *Mc. marinus* can be detected and monitored by MRI because of changes in the magnetic field caused by magnetosomes [139].

Isolated magnetosomes, especially when functionalized with proteins or antibodies, have an even broader range of applications than intact living or dead cells of MTB, including bioremediation, separation of molecules, drug carriage, gene therapy, and cancer treatments. As stated above, isolated magnetosomes can be highly purified and sterilized. Therefore, biomedical applications using magnetosomes are likely safer when used in living cells or organisms. Many potential medical and environmental applications of isolated magnetosomes have been examined. Ginet *et al.* (2011) [85] showed that the magnetosomes can be used as reusable biocatalysts. When functionalized with the enzyme phosphohydrolase, they can be used to degrade certain pesticides since the catalytic function of the enzyme after expression in the surface of the magnetosome membrane is preserved. These modified magnetosomes can be recycled and the catalytic activity of the phosphohydrolase, in one study, remained stable after repeated cycles of degradation of the pesticide [85]. Isolated magnetosomes from MTB genetically modified to express a multi-subunit enzyme complex of a chimeric RNaseP in magnetosomes showed full RNaseP activity [129]. Magnetosomes functionalized with the enzyme PPDK (pyruvate phosphate dikinase) proved usful in pyrosequencing. The magnetosome-enzyme display system could also be recycled without major loss of enzyme activity and magnetic separation allowed for a rapid buffer exchange, stringent washing, and reduced non-specific binding. These features reduced costs and improved the analytical process [140].

The identification of minimal quantities of targeted molecules can be determined using Immuno-PCR technology. In this case, the targeted molecule is recognized by antibodies and then marked with DNA for RT-PCR. A variant of this assay with antibody-functionalized magnetosome (MagnetoImmuno-PCR) allows immobilization of the targeted molecule and its magnetic separation. This technique can be used independently from solid phase materials and retain all the advantages from standard Immuno-PCR [141]. Matsunaga and collaborators (2007) built complexes of magnetosome with polystyrene microbeads that provided a larger interaction surface. The complexes were more efficient than the magnetosomes alone when applied to automated immunoassay for the detection of human prostate-specific antigen (PSA) [142]. Magnetosomes conjugated with antibodies consist of a sensitive tool for magnetic separation and specific screening of target cells. Magnetosomes remain connected when the cells are placed again in culture and do not appear to interfere with growth and differentiation. In addition, the magnetosomes do not interfere with spectrophotometric measurements which can be a problem when artificial magnetic particles are used [143]. Magnetosomes have been used as markers of biomolecular interactions using magnetic force microscopy (MFM). For example, streptavidin molecules, immobilized on a slide, bind to magnetosomes conjugated with biotin which can be identified using MFM, resulting in an assay that is 100 times more sensitive than the streptavidin detection with fluorescence [144].

Magnetosomes have been used as carriers of specific molecules in many studies. It has been shown that magnetite magnetosomes attach to a larger amount of drug molecules in cancer chemotherapy than artificial, chemically-synthesized magnetite particles and provide more control over the drug release [145]. A good example of this is the drug doxorubicin [112]. Magnetosomes injected into the tail vein of nude mice localized mainly in the liver and lungs but not in other organs. They could be detected even after 2 weeks and, therefore, suggesting that they can be used to deliver drugs specifically to these organs [146].

Magnetosomes have also been used as carriers of recombinant DNA to produce a genetic vaccine for the immunotherapy of tumors. Treated tumors showed a significant reduction and no apparent toxicity to the vaccinated mice. Moreover, magnetosomes showed no immunogenicity: an immune response was induced only by pieces of DNA bound to the magnetosome demonstrating that magnetosomes have great promise as carriers for technologies involving gene therapy and genetic immunization [147]. Magnetosome cytotoxicity is apparently low as the viability of cells incubated with magnetosomes after 72 h was 90%. The MM of the magnetosome seems to provide better biocompatibility than synthetic magnetite [148].

Magnetosomes can also be used directly for cancer treatment using hyperthermia therapy. Magnetosomes placed in tumor tissues are exposed to an external, alternating magnetic field thereby releasing heat that theoretically kills tumor cells and thus eliminates or decreases the size of the tumor [37]. It is noteworthy that chains of magnetosomes were more effective in killing tumor cells in comparison to individualized magnetosomes. The acute toxicity of magnetosomes injected into rats appears to be low but further studies accessing the risk-benefit of this treatment are necessary [86]. Where magnetosomes accumulate after being injected into the bloodstream of an organism is still a matter of debate. Little data is available and they indicate magnetite magnetosomes and chemically-synthesized magnetite particles differ in their destination in the organism [123]. Some magnetosomes administered to mice were found in lysosomes of their liver and spleen suggesting that macrophages remove magnetic particles from the bloodstream and carry the magnetosomes to these organs [149,150]. Particles in these lysosomes were partly digested and no particles were found in the faeces or urine of those animals [149] although there is one report of magnetosomes being eliminated from mice via feces [86]. Additional studies are clearly warranted to determine the distribution and elimination of magnetosomes in organisms treated with these particles.

As new strains of MTB are isolated, more of their genomes become available, the functions of more magnetosome proteins are elucidated, and new molecular techniques are developed, it is likely that the numbers of commercial, scientific, and biomedical applications of magnetosomes and bioinspired magnetic nanoparticles will increase and become more widespread especially as economically viable strategies emerge for the production of these structures. Applications of magnetosomes as drug carriers, as contrasting agents or in cancer therapy clearly will require regulation of the US Food and Drug Administration (FDA) and other similar agencies. Two main aspects must be addressed in order to achieve FDA requirements: the Guidelines for Manufacturing Practices (GMP) and tests for the toxicity or side effects of the treatment, such as Limulus Amoebocyte Lysate (LAL) tests applied for other drugs [151]. Although a specific guideline for the use of magnetosomes in medical treatments does not exist yet, it may be possible that the guidelines for the approval of chemically-synthesized magnetic particles for use as contrasting agents in MRI and in cell separation assays [152] could be applied to isolated magnetosomes.

## 3. Genomic Studies on MTB as Potential Sources for New (Natural) Products

The increasing availability of complete and incomplete genome sequences of MTB has greatly improved the culturing of these fastidious microorganisms by providing information of their metabolic capabilities and of their nutritional requirements [153]. Genomic data, in general, has helped to determine an organism’s metabolism by revealing which genes are present and may be involved in specific biochemical pathways. This cannot always be determined in laboratory growth experiments. Thus genomic information is also helpful for elucidating appropriate substrates for growth and/or optimization of growth media that will support the isolation and maintenance of new strains [6,154] and enhance the expression of genes coding for bioproducts of interest.

We have used a genome mining strategy in marine MTB to detect genes encoding secondary metabolites and other bioproducts that might be able to be co-produced in bioreactors once the proper conditions for optimal gene expression are determined. We applied the antiSMASH platform [155], which searches whole genomes or contigs for conserved domains in gene clusters coding for natural product biosynthesis. This platform uses profile Hidden Markov Models (pHMMs) to search query protein-encoding genes for signatures or protein domains that have been experimentally accessed. Homologies are further investigated with implemented ClusterBlast analyses that consider as match those with a minimum specified value (*e*-value < 1E-05; 30% minimal sequence identity and coverage >25%) [155]. Secondary metabolites comprise an important class of pharmaceutically active compounds that includes antibiotics, antiparasitics, immunosuppressants, and anti-cancer drugs. Most of these types of metabolites have been found to be produced by species of the Domain *Bacteria* and the Kingdom *Fungi* isolated from different environments [156]. Strains of the prokaryotic genus *Streptomyces* have been regarded as the greatest source of these types of active compounds but recent genome studies have revealed that many species, including anaerobic prokaryotes, contain clusters of genes involved in secondary metabolite synthesis [156,157]. The main pathways for the synthesis of those compounds are the nonribosomal peptide synthetase (NRPS) and polyketide synthase (PKS) pathways. Three classes of PKS systems exist and, although their mechanism of function differs slightly, they are involved in the production of a variety of complex molecular structures by the oligomerization of simpler molecules [158]. PKS I is the most studied class and consists of large proteins containing different active domains that include acyltransferases, acyl-carrier proteins, dehydratases, ketosynthases and ketoreductases. NRPS systems are also divided into enzymatic domains such as adenylation, thiolation, condensation, epimerization, methylation, reduction, and cyclisation [158]. This system selects and modifies amino acids to generate molecules of interest [156]. Both pathways can be expressed together and generate PKS-NRPS hybrid products, enhancing the diversity of bioactive compounds produced through these systems [158].

As discussed in an earlier section, all known MTB from aquatic environments, ranging from brackish to hypersaline, phylogenetically belong to the phylum *Proteobacteria* while some freshwater strains belong to the *Nitrospirae* phylum and PVC superphylum as well as the *Proteobacteria*. PKS and NRPS domains have been found in both aerobic and anaerobic members of the *Proteobacteria* [156,157] but, to our knowledge, the presence of these domains has never been investigated in MTB. There are currently seven completely sequenced genomes of MTB publicly available including that from: the marine strains *Magnetococcus marinus* [57], *Magnetospira* sp. QH-2 [56] and *Candidatus* Magnetoglobus multicellularis [153]; the freshwater sulfate-reducing bacterium *Desulfovibrio magneticus* [159] and *Magnetospirillum* *magneticum* strain AMB-1 [160], *Ms. gryphiswaldense* strain MSR-1 [161] and *Magnetospirillum* sp. strain SO-1 [162]. Other genomic data discussed in this work are from MTB genomes currently under assembly or annotation and have not been published yet. In our analyses (Table 4), we found clusters of genes with conserved domains from PKS and NRPS in MTB which might represent a yet unexplored source for new bio-compounds.

With regard to the cultured magnetococci, we did not detect PKS or NRPS domains in the genome of *Magnetococcus marinus* as previously reported [57] and found only three ORFs (open reading frames) containing PKS-NRPS domains in the genome of *Magnetofaba australis*. These were organized as a unique gene cluster flanked by transposase genes typical of a genomic island [163]. All ORFs presented higher similarity to genes sequenced from the Gram-positive, spore-forming bacterium *Paenibacillus curdlanolyticus* (identity ranging from 40% to 51%, coverage from 89% to 99%) and a much higher GC content (63.71% to 68.60%) than the rest of the *Mf. australis* genome (57.98%) indicating that they were probably transferred horizontally to the magnetotactic strain. Therefore, we do not believe that the cultured magnetotactic cocci would be good candidates for searches involving the production of novel secondary metabolites. The same applies to the marine spirillum *Magnetospira* strain QH-2, whose genome appears to contain only two ORFs containing NRPS domains, both with low similarity to amino acid adenylation enzymes from cyanobacteria (identity 40%, coverage 87%).

**Table 4:** Number of ORFs containing polyketide synthase (PKS) and/or nonribosomal peptide synthetase (NRPS) conserved domains in analyzed magnetotactic genomes.

Species	Strain	Class ^†^	Source	Salinity	Genome (Mb)/MAI (Kb)	PKS	NRPS	Hybrid
*Mc. marinus*	MC-1	α	Pettaquamscutt Estuary—USA	Brackish to marine	4.71/55.82	0	0	0
*Mf. Itaipuensis* *	IT-1	α	Itaipu lagoon—Brazil	Brackish to marine	4.98/64.9	3	0	0
*Mv. blakemorei*	MV-1	α	Saltmarsh pool—USA	Brackish	3.70/66.03	6	0	1
*Magnetospira* sp.	QH-2	α	Intertidal seawater—China	Saline	4.0/45	0	2	0
*Ms. gryphiswaldense*	MSR-1	α	Eutrophic river—Germany	Freshwater	4.36 + 0.036/74.6	3	0	2
*Magnetospirillum* sp. *	SO-1	α	River—Russia	Freshwater	4.87/100	0	0	1
*Ms. magneticum*	AMB-1	α	Koganei ponds—Japan	Freshwater	4.97/73	0	0	1
Order *Chromatiales*	SS-5	γ	Salton Sea—USA	Hypersaline	3.7/ND	1	3	5
*Ca.* Da. Magnetomortis *	BW-1	δ	Badwater Basin—USA	Brackish	6.8/ND	8	4	3
*Ca.* Mg. multicellularis *	MMP	δ	Araruama Lagoon—Brazil	Hypersaline	12.8/15.7	9	11	4
*Desulfovibrio magneticus*	RS-1	δ	Kameno River—Japan	Freshwater	5.25 + 0.058 + 0.008/71	0	0	0

^†^ All marine strains of magnetotactic bacteria belong to phylum *Proteobacteria*. * Genome assembly of these species is not complete and the number of ORFs (Open Reading Frames) might be reduced in the final analysis.

In contrast, the genomes of the unnamed gammaproteobacterial strains SS-5 and BW-1 contain a relatively large number of PKS and/or NRPS domain-containing ORFs, 9 and 15, respectively (Table 4). However, these genomes are not completely assembled and therefore the number of ORFs may be slightly reduced or increased and the arrangement of gene clusters and genes within clusters might differ in the final assembly. In the genome of *Candidatus* Desulfamplus magnetomortis strain BW-1, we discovered a 4210 amino acid ORF with PKS domains similar to genes found in members of the family *Desulfobacteraceae* (coverage 96%, identity 62%), the family most closely related phylogenetically to strain BW-1. Another gene cluster is present that contains 12 ORFs with hybrid domains that show low similarity to genes encoding proteins with PKS and NRPS domains (coverage ranging from 85% with identity of 39% to coverage of 84% and identity of 62%) although no specific biosynthetic pathway could be assigned to them. The larger ORF in strain SS-5 (7595 amino acids) indicates some similarity to putative PKS-NRPS proteins from *Streptomyces* species (identity 38%, coverage 90%). In another gene cluster, a 5128 amino acid ORF best matched the *ttcB* gene from the marine *Thalassospira* sp. (identity 44%, coverage 99%), which is involved in the synthesis of thalassospiramide, a compound with immunosuppressant activity [164]. A third gene cluster contains an ORF with NRPS domains most similar to the *nosD* gene (identity 39%, coverage 95%), part of the nostopeptolide gene cluster originally discovered in the cyanobacterium *Nostoc* sp. [165]. Two other ORFs in a fourth cluster (2163 and 1321 amino acids long) were also similar to genes encountered in cyanobacteria (coverage 98%, identity 29%; coverage 99%, identity 38%, respectively). The low level of similarity between genes containing PKS-NRPS and characterized proteins denotes the importance of the search for conserved sites in these multi-domain proteins [157]. The putative function of these proteins remains unknown given their low similarities to described proteins; however, the search for conserved domains in a cluster of ORFs permitted their assignment to secondary metabolite pathways. The differences between the described proteins and the new ORFs analyzed in this work represent the potential of new bioactive molecules produced by MTB and reinforce the need of further studies to characterize their expression, especially in new organisms in which secondary metabolite pathways remain unexplored.

The genome of the freshwater magnetotactic bacterium *Magnetospirllum magneticum* contains a single ORF containing hybrid domains, whereas five ORFs with PKS and NRPS domains were detected in *Ms. gryphiswaldense*, suggesting that modification of the growth medium or culture conditions for the later bacterium might provide the necessary environment and substrates for the enhanced expression of genes encoding secondary metabolites. Although searches in the genomes of the deltaproteobacterial MTB strains are not conclusive because most of the genomes available are not closed, the relatively high amount of secondary metabolite gene domains found suggests these organisms might have a relatively complex metabolism as previously indicated by culture and genomic studies [24,153]. MTB of the *Deltaproteobacteria* class have unique features in comparison to other MTB. This is the only class in which greigite synthesis was detected in MTB and includes all forms of the multicellular MTB and strains capable of biomineralizing both greigite and magnetite in their magnetosomes (e.g., *Candidatus* Desulfamplus magnetomortis strain BW-1). The complex metabolic mechanisms involved in the regulation of these features might be related to the presence of a large number of ORFs containing PKS and NRPS domains within the genomes of these MTB. Among the magnetotactic *Deltaproteobacteria* is also the freshwater, sulfate-reducing bacterium *Desulfovibrio magneticus* which synthesizes bullet-shaped magnetite magnetosomes [23]. Although a high number of transposable elements are present in its genome [159], we could not identify ORFs containing PKS or NRPS domains, indicating that this bacterium does not have a strong potential for the production of secondary metabolites.

**Figure 7:**
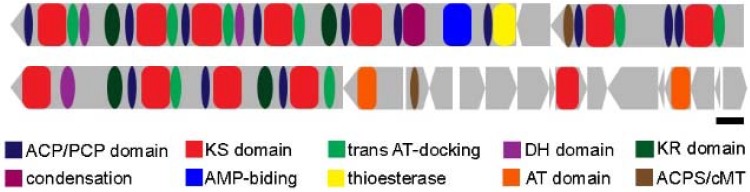
Cluster of genes containing PKS-NRPS domains in the genome of *Magnetovibrio blakemorei* strain MV-1. Scale bar = 1 Kb. ACP/PCP—acyl carrier protein/peptidyl carrier protein; KS—ketosynthase; AT—acyltransferase; DH—dehydratase; KR—β-ketoreductase; AMP—adenosine monophosphate; ACPS/cMT—acyl carrier protein synthase/c-methyl transferase.

The most promising marine magnetotactic candidate for the production of secondary metabolites appears to be *Magnetovibrio blakemorei* strain MV-1 which is the most metabolically versatile magnetotactic bacterium [17] and has been mass cultured to large scale [39]. A large gene cluster containing seven ORFs with PKS-NRPS domains (Figure 7) is present in the genome of *Mv. blakemorei*. The longest ORF (6560 amino acids) in this cluster encodes a multi-domain protein whose highest degree of similarity is to a protein of unknown function present in *Ms. gryphiswaldense* (identity 51%, coverage 98%). Proteins encoded by two other ORFs (4345 and 2570 amino acids) in the same cluster are similar to beta-ketoacyl synthase found in *Ms. gryphiswaldense* MSR-1 (identity 51%, coverage 98%) and *Oceanibaculum indicum* (identity 55%, coverage 96%), respectively. The four smaller ORFs in the cluster include genes that encode for beta-ketoacyl synthase and malonyl CoA-acyl carrier protein, also similar to proteins from *Ms. gryphiswaldense* and *O. indicum* (similarity higher than 58% and coverage 98%). It is noteworthy that *Magnetospirillum*, *Magnetovibrio*, and *Oceanibaculum* all phylogenetically belong to the family *Rhodospirillaceae* suggesting that this gene cluster has been in the genomes of these organisms for some time and is stable in the genome. Considering that both *Ms. gryphiswaldense* and *Mv. blakemorei* are already being mass cultured in large scale for the production of magnetosomes, this cluster of genes deserves further attention. Elucidation of pathways and products in these MTB could lead to the co-production of new bioactive molecules in conjunction with magnetosomes.

## 4. Conclusions

Magnetotactic bacteria are ubiquitous in marine and freshwater sediments and biomineralize magnetosomes, magnetic nanocrystals of magnetite or greigite enveloped by a lipid bilayer derived from the cytoplasmic membrane. This ability to synthesize magnetosomes has stimulated and motivated a great deal of research involving diverse commercial, scientific and biomedical applications of MTB that require or could be improved using magnetic nanocrystals. The synthesis of magnetosomes is genetically controlled and results in the biomineralization of single magnetic domains: permanent magnetic crystals that have a high degree of crystallographic perfection and consistent sizes and morphologies. Because of the magnetosome lipid bilayer, the crystals are biocompatible and can be modified in numerous ways. These characteristics are important in biotechnological applications of magnetic nanoparticles and are not all generally obtained in chemically-produced magnetic nanocrystals. Major drawbacks to the widespread application of magnetosomes involve the fastidiousness of MTB regarding growth, which makes them difficult to culture on a large scale, and the need to fully understand the genetic/environmental control over magnetosome synthesis. However, these limitations are constantly being addressed by numerous researchers. The description of MTB as well as the increasing availability of sequenced genomes contributes to the optimization and scaling of magnetotactic cell cultivation. Moreover, economic feasibility of magnetosome production in large scale might be achieved through the co-production of magnetosomes and other metabolic products of high added-value. The marine magnetotactic strain *Magnetovibrio blakemorei* and the freshwater *Magnetospirillum gryphiswaldense* are amenable to cultivation in large scale and their genomes clearly reveal their potential to produce secondary metabolites under as yet unknown conditions. The combination of genomic and growth studies on MTB are necessary to overcome the difficulties currently inherent in handling MTB and to create an economically viable production of magnetosomes.
